# Left Anterior Descending Artery to Pulmonary Artery Fistula: A Case Report

**DOI:** 10.7759/cureus.26713

**Published:** 2022-07-10

**Authors:** Varun George, Silvan Omerovic, Mohan Madala, Awais Kang

**Affiliations:** 1 Internal Medicine, McLaren Greater Lansing, Lansing, USA; 2 Cardiology, McLaren Greater Lansing, Lansing, USA; 3 Interventional Cardiology, McLaren Greater Lansing, Lansing, USA

**Keywords:** non-st segment elevation myocardial infarction (nstemi), st-elevation myocardial infarction (stemi), : acute coronary syndrome, stable angina, coronary artery fistula (caf)

## Abstract

This case describes a 59-year-old patient who initially presented with symptoms consistent with stable angina and a subsequent diagnosis of non-ST elevation myocardial infarction with further conversion to an ST-elevation myocardial infarction. A left cardiac catheterization was scheduled to evaluate the patient’s acute coronary syndrome. He later developed worsening chest pain and a repeat electrocardiogram (ECG) showed ST elevations in anterolateral leads. The patient was emergently transported to the cardiac catheterization lab. The coronary angiogram revealed a proximal left anterior descending artery (LAD) lesion. During the catheterization, abnormal communication between the LAD and the pulmonary artery was discovered and the patient was diagnosed with a coronary artery fistula. This case presents a unique scenario for an ST-elevation myocardial infarction with an incidental diagnosis of a coronary artery fistula.

## Introduction

Coronary artery fistulae (CAF) are rarely described as congenital malformations that result in abnormal communication between coronary vessels and a portion of the cardiac chambers or pulmonary vasculature. The precise incidence of the abnormality is unknown, however, an estimated 0.3% of cardiac abnormalities are known to be due to CAF [1]. A fistula between the left anterior descending artery (LAD) and pulmonary artery is the rarest variant reported and comprises approximately 17% of all CAF cases. While most common CAF connects from the LAD to the right ventricle which is described in approximately 41% of cases [1]. These abnormalities may arise from either congenital defects, trauma to the anterior thorax secondary, or previous cardiac surgeries [1]. While most cases are asymptomatic and are discovered incidentally, symptomatic patients have been shown to present with symptoms of cardiac ischemia or heart failure. Due to the incidental discovery of CAF in the majority of patients, the actual incidence of CAF in the general population is likely underreported and less frequently categorized [2]. Treatment of the fistula is indicated in patients who experience symptoms to prevent future complications that can arise from significant shunting of coronary flow with transcatheter and surgical techniques both being utilized for closure [2]. 

## Case presentation

**Figure 1:**
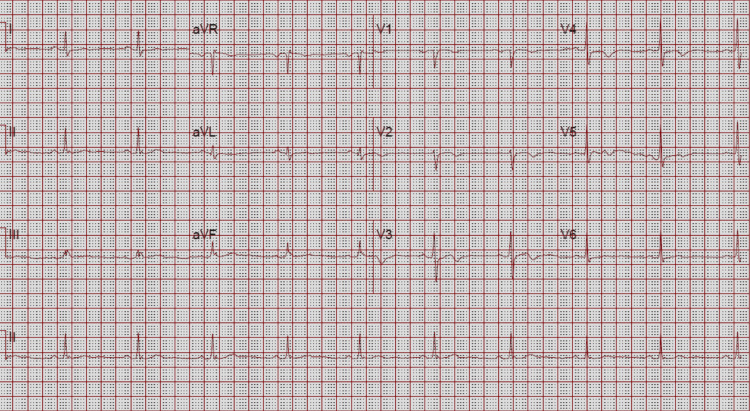
Initial electrocardiogram that shows sinus bradycardia with noted biphasic T wave in V3 and T wave inversions in leads V4 and V5.

**Figure 2:**
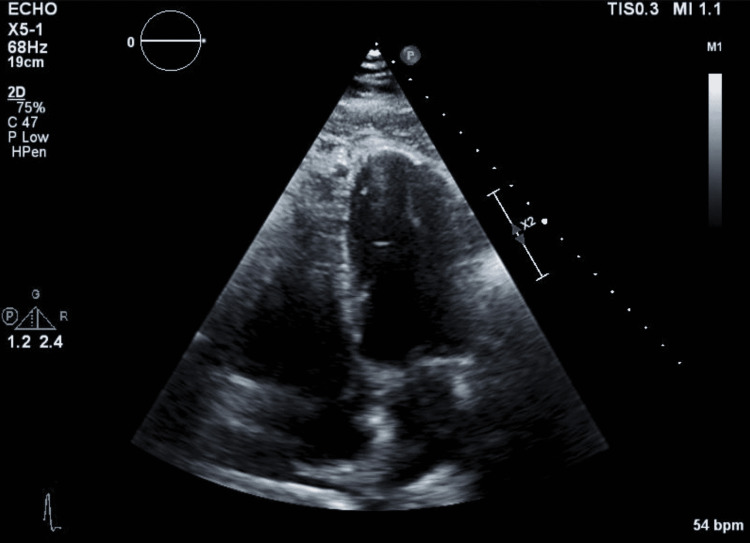
Transthoracic echocardiogram, apical four chamber view.

**Figure 3:**
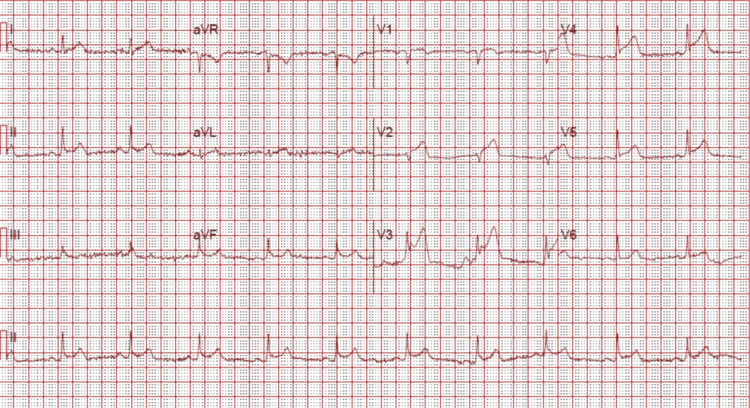
Electrocardiogram that shows sinus bradycardia with greater than 2 mm ST-segment elevations in leads V2, V3, V4, and V5 signifying antero-lateral ischemia.

**Figure 4:**
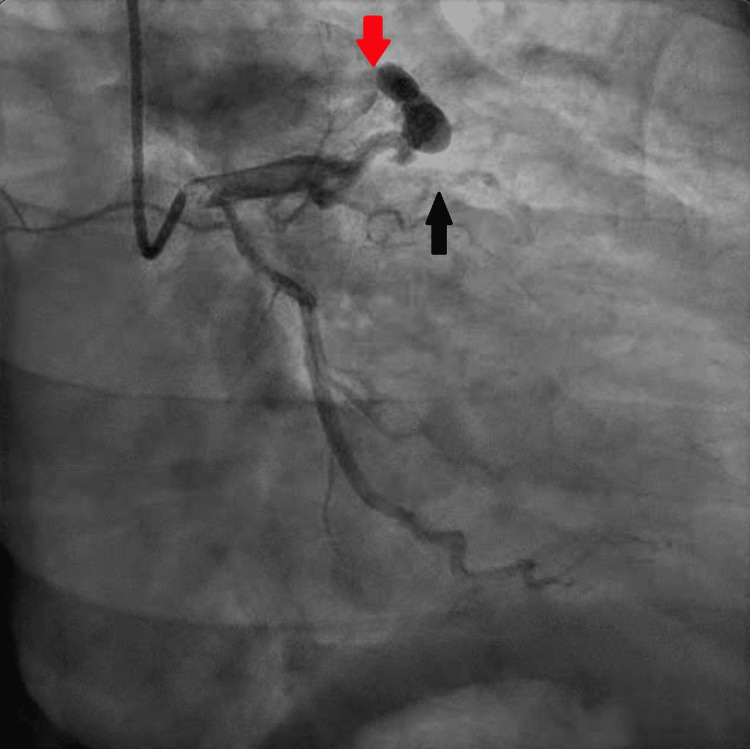
Left coronary angiography with a RAO caudal view after initial engagement of the left main coronary artery. Proximal LAD occlusion noted (black arrow) with a LAD to pulmonary artery fistula visualized (red arrow). Right arrow, coronary artery fistula; black arrow, occluded LAD; RAO, right anterior oblique; LAD, left anterior descending artery

**Figure 5:**
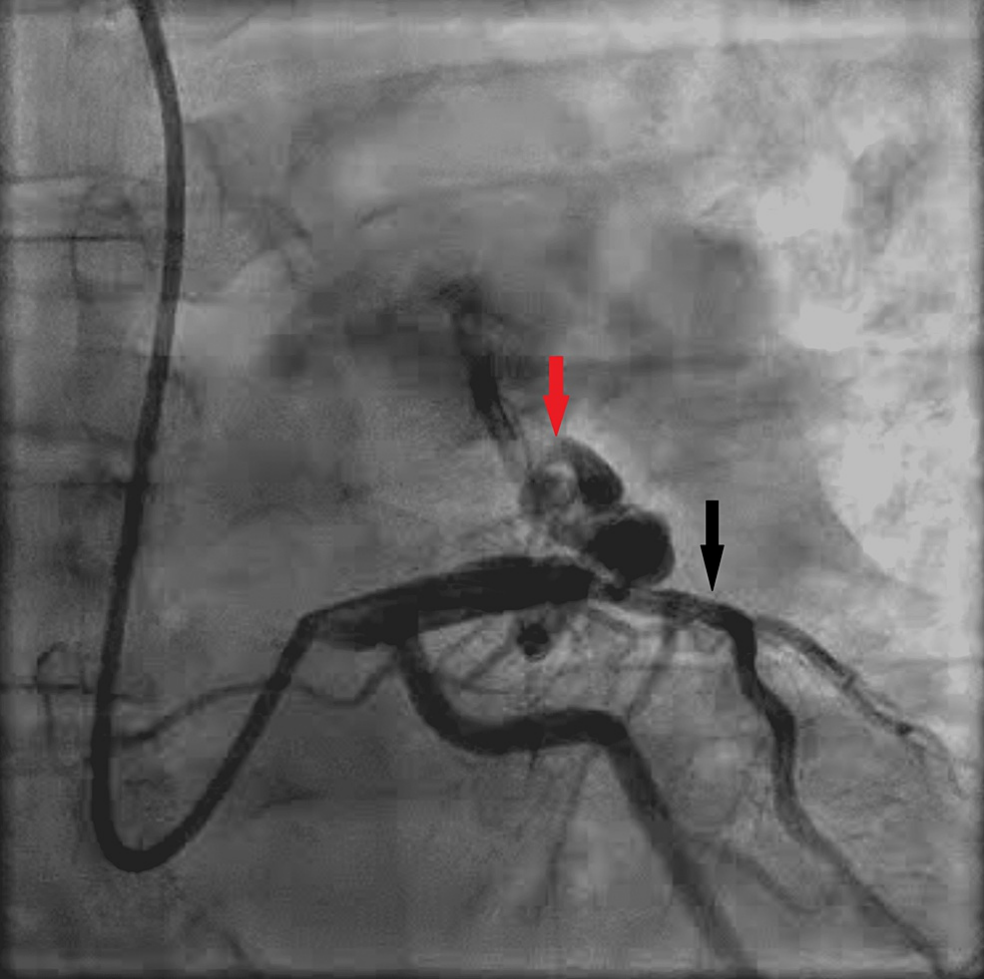
LAO caudal view of the LAD after PTCA and LAD stent placement. Patent LAD after stent (black arrow) with continued visualization of LAD to pulmonary artery fistula (red arrow). Red arrow, left anterior descending artery to pulmonary artery fistula; black arrow, patent left anterior descending artery after drug-eluting stent; LAD, left anterior descending artery; LAO, left anterior oblique; PTCA, percutaneous transluminal coronary angioplasty

A 59-year-old patient with a known medical history of and hypertension and pre-diabetes initially presented with symptoms of chest pain that increased with exertion and was relieved with rest. These symptoms worsened and the patient developed pain with very minimal exertion with radiation to the neck. The initial electrocardiogram (ECG) showed sinus bradycardia with a heart rate of 59 beats/min, a normal axis, with T wave inversions noted in leads V2 through V5 and additional biphasic T waves noted in lead V3 (Figure 1). Initial high sensitivity troponin I was elevated at 68.6 ng/L, which increased to a peak of 90.8 ng/L prior to the cardiac catheterization. The transthoracic echocardiogram showed a left ventricular ejection fraction of 60%-65% with moderate septal wall hypertrophy (Figure 2). The diastolic filling was noted to be similar to age-related diastolic function. There was diffuse sclerosis of the aortic valve cusp with no stenosis. Right ventricular systolic function was at the upper normal at 35 mmHg. The patient was evaluated by the cardiology team and was diagnosed with a non-ST elevation myocardial infarction (NSTEMI) and recommended ischemic evaluation with a coronary angiogram. The patient was started on a heparin infusion and given a loading dose of 324 mg of aspirin during the initial evaluation. The patient was also started on aspirin 81 mg once daily, atorvastatin 40 mg once daily, and metoprolol tartrate 12.5 mg once daily. In the next 24 h of the hospital course, the patient developed worsening chest pressure and a repeat ECG revealed significant ST elevations in leads V2, V3, V4, and V5 (Figure 3). The patient was subsequently transferred to the cardiac catheterization laboratory and a coronary angiogram was performed. A 6-French extra backup (EBU) guide catheter was engaged into the left main coronary artery. During the cardiac catheterization, a fistula from the LAD into the pulmonary artery was identified prior to the percutaneous transluminal coronary angioplasty (PTCA) (Figure 4). Initial engagement of the left main coronary artery with an injection of the contrast agent also revealed the abnormal movement of the contrast to a larger vessel (Figure 4). Further examination shows a fistula directly from the LAD to the pulmonary artery (Video 1). The LAD lesion was further evaluated and noted to be distal to the coronary artery fistula. A wire was placed in the distal LAD and a 2.5 mm x 15 mm compliant balloon was used for pre-dilation of the proximal LAD. The lesion required a drug-eluting stent 3.0 mm x 28 mm in size which was post-dilated with a 3.5 mm x 12 mm non-compliant balloon at 20 atm of pressure (Figure 5). No evidence of the coronary steal phenomenon was noted during coronary angiography. Angiographic films after PTCA and stent placement reveal a patent LAD and continue to show the large CAF to the pulmonary artery (Video 2). The patient was discharged with dual anti-platelet therapy consisting of aspirin 81 mg daily along with ticagrelor 90 mg twice daily, atorvastatin 80 mg daily, and metoprolol tartrate 12.5 mg daily. A cardiac magnetic resonance image (C-MRI) will be performed to evaluate the fistula for possible coiling or surgical intervention.

**Video 1:** RAO caudal view during initial engagement of the left main coronary artery showing a proximal LAD lesion and a LAD to pulmonary artery fistula. RAO, right anterior oblique; LAD, left anterior descending artery

**Video 2:** LAO caudal view of LAD artery revealing abnormal connection from LAD to pulmonary artery after PTCA and stent placement and restoration of circulation to the LAD. LAD, left anterior descending artery; LAO, left anterior oblique

## Discussion

Coronary artery fistulae are either congenital or acquired abnormal vascular communications that are generally described as a connection between the systemic and pulmonary circulation of the cardiac vasculature [3]. Coronary artery abnormalities generally account for a very small percentage of congenital heart diseases; noted to be approximately 0.3% of all congenital cardiac abnormalities. Generally, CAF is described with a triad of findings including a potential cardiac murmur, a large tortuous coronary artery, and an atrial or ventricular left to right shunt. Communication from the LAD to the pulmonary artery has the lowest incidence of 17% in the general population [1]. 

Coronary artery fistulae can be further categorized as coronary-cameral fistula or coronary IV fistula. A coronary cameral fistula is an abnormal communication between a coronary artery and one of the cardiac chambers. While a coronary intravenous fistula is noted to be a connection from the coronary artery to the systemic or pulmonary circulation [3]. This abnormality can be a congenital malformation or acquired secondary to trauma or cardiac bypass surgery. CAF can cause symptoms in patients when left untreated, with a higher incidence of symptoms in older patients. Common sequelae of untreated CAF may include myocardial ischemia, cardiomyopathy, congestive heart failure, and pulmonary hypertension. While most cases of CAF are incidental findings in patients, most older patients have a sequela of coronary artery diseases as the primary presenting illness [4].

Precise evaluation of CAF is accomplished via conventional coronary angiography. Coronary angiography will also aid in further diagnosis and possible options for embolization. In addition, transthoracic or transesophageal echocardiography can be used to evaluate the anatomy of CAF [5]. The use of microbubble contrast material may enhance the location of the CAF, however, this has limited utility and cannot aid in the evaluation of any possible occlusions or collateral vessels. Additionally, distal segments of the CAF may be lost with this technique. Coronary CT angiography can also be used for further evaluation of the precise CAF anatomy [5]. The model rendering of CT angiography of anatomical landmarks including the origin and course of the CAF can be more precisely located. CT angiography, however, does have inherent radiation exposure which should be considered in imaging recommendations. C-MRI can also be used as an alternative to conventional or CT angiography. While C-MRI has benefits in patients that may require multiple images due to the lack of radiation exposure or contrast material, in comparison to CT angiography, there is decreased resolution of the CAF and less clear anatomy [5].

Further management of CAFs can consist of both medical management and surgical closure of the fistula. Closure of large fistulae is currently recommended by The American College of Cardiology and American Heart Association guidelines. For patients who present with small to moderate fistulae, closure is recommended for symptomatic patients [3]. All patients with CAF should have periodic echocardiographic follow up every three to five years to evaluate changes in the size of the fistula. Additionally, patients who have no evidence of shunting can be managed medically with optimal anti-anginal therapy. The treatment of CAF can be via catheter-based coil occlusion or surgical ligation of the fistula [6]. Surgical closure can also be performed with or without cardiopulmonary bypass techniques [7].

This case specifically describes a LAD communicating to the pulmonary artery and trunk. This was an incidental finding during a left cardiac catheterization that was performed due to an ST-elevation myocardial infarction. This patient had no prior symptoms from his CAF that were noted and additionally he had no prior history of chest trauma or cardiac surgery. This patient’s CAF is likely congenital in nature and did not previously cause any abnormalities in cardiac circulation to note, however, it is difficult to ascertain the precise etiology due to the incidental nature of discovery. To fully evaluate the degree of the size of the fistula, a CT angiography or C-MRI will need to be completed to assess the method of closure that would be appropriate. As this patient initially presented with symptoms of typical angina and was subsequently diagnosed with acute coronary syndrome and myocardial infarction, a closure of the fistula is the most appropriate course of management. This patient's case shows unique anatomical abnormalities and coronary circulation that require further management beyond coronary artery disease after PCTA and drug-eluting stent. The evaluation of the coronary artery fistula with the size degree of shunting will likely prevent further complications including worsening coronary disease as well as the potential cardiomyopathy in this patient [4].

## Conclusions

This patient case presents a unique anatomical abnormality of an incidentally discovered coronary artery fistula during a left cardiac catheterization and coronary angiography. The fistula shows abnormal communication between the LAD and the pulmonary artery. It is unclear if this patient’s anatomical abnormality causes a predisposition to coronary artery disease as this link has not been established. However, as most patients with CAF are asymptomatic, angina has been described in patients who have an incidental CAF. This patient’s presenting symptom was addressed during the cardiac catheterization as a significant flow-limiting lesion in the LAD was treated with a drug-eluting stent. The case describes a rare cardiac abnormality and outlines the evaluation and treatment to ensure appropriate management for this patient.
